# Relationship between preterm, low birth weight, and development defects of enamel in the primary dentition: A meta-analysis

**DOI:** 10.3389/fped.2022.975340

**Published:** 2022-11-10

**Authors:** Shan Xu, Caiyun Zhao, Liying Jia, Zhe Ma, Xiaolin Zhang, Hong Shi

**Affiliations:** ^1^ Department of Pediatric Dentistry, Hospital of Stomatology & Hebei Provincial Key Laboratory of Stomatology, Hebei Medical University, Shijiazhuang, China; ^2^ Department of Preventive Dentistry, Hospital of Stomatology & Hebei Provincial Key Laboratory of Stomatology, Hebei Medical University, Shijiazhuang, China; ^3^ Department of Epidemiology and Statistics, School of Public Health, Hebei Province Key Laboratory of Environment and Human Health, Hebei Medical University, Shijiazhuang, China

**Keywords:** preterm, low birth weight, developmental defect of enamel, primary dentition, meta-analysis

## Abstract

**Background:**

This study aimed to systematically analyze the relationship between preterm (PT), low birth weight (LBW), and developmental defects of enamel (DDE) in the primary dentition.

**Methods:**

Following the retrieval of the databases, case–control studies, cross-sectional studies, and cohort studies on the relationship between PT, LBW and DDE, which had been published in English or Chinese up to January 2022 were included. The data about odds ratio (OR) and 95% confidence interval (95% CI) were extracted and calculated using STATA 12.0 Software. Case–control studies were evaluated using the Newcastle–Ottawa Scale (NOS), while cross-sectional studies and cohort studies were evaluated using the JBI scale. The heterogeneity of each study was evaluated using the Q test.

**Results:**

A total of 15 studies were included, of which 8 studied the relationship between PT and DDE, and 13 explored the relationship between LBW and DDE including three about the relationship between very low birth weight (VLBW) and DDE. Seven studies explored the relationship between PT, LBW, and DDE. The results of this meta-analysis showed that both PT and LBW especially VLBW (OR^ ^= 7.19, 95% CI: 4.98–10.38) were risk factors for DDE in the primary dentition (OR^ ^= 2.33, 95% CI: 1.55–3.51) (OR^ ^= 1.67, 95% CI: 1.08–2.59). The subgroup results showed that PT and LBW were both associated with the occurrence of enamel hypoplasia (EHP) (OR^ ^= 6.89, 95% CI: 3.33–14.34; OR^ ^= 2.78, 95% CI: 2.10–3.68) rather than enamel opacity (OR^ ^= 0.94, 95% CI: 0.55–1.61; OR^ ^= 1.03, 95% CI: 0.66–1.61). There was no publication bias about the included studies (*P* = 0.75 > 0.05; *P* = 0.47 > 0.05).

**Conclusion:**

This meta-analysis demonstrated that both PT and LBW especially VLBW are associated with a higher risk of DDE in the primary dentition. PT and LBW are both related to the occurrence of EHP. However, the relationship between PT, LBW, and enamel opacity has not been verified.

**Systematic Review Registration:**

https://www.crd.york.ac.uk/prospero/display_record.php?, identifier: CRD42021262761.

## Introduction

During odontogenesis, many factors can affect the ameloblastic function and interfere with the enamel organ formation process, triggering anomalies called developmental defects of enamel (DDE) in the primary dentition (1, 2). According to epidemiological studies, DDE occurs in 15%–49% of primary dentitions worldwide in the last two decades (3–7). Previous studies showed that plaques can easily adhere to the pits and spots on the tooth surface caused by DDE, which increases the incidence and enhance the progression of early childhood caries (ECC) (7, 8). The influence of ECC on DDE children is great and quick. As a result, DDE in primary dentition poses a significant risk of ECC, and lowering its prevalence can lessen the impact of ECC on children's physical and mental health (9).

The etiology of DDE is diverse. The formation of primary dentition starts during pregnancy and ends after the birth of the child, during which there are various maternal and infant risk factors affecting ameloblasts and odontoblasts (10). The global or local effects lead to qualitative or quantitative defects in the enamel during the formation, mineralization, and maturation of the enamel matrix (1). DDE is frequently thought to be influenced by a variety of factors, including genetic disorders, the mother's physical health throughout pregnancy, fetal intrauterine infection, the maternal state at birth, and the infant's condition after birth (11). Most studies focus on the physical condition of mothers during pregnancy, low birth weight (LBW) or preterm (PT) during birth and the infant's condition after birth (10). Many studies showed that an infant's condition after birth such as PT, and LBW may be related to DDE (10, 11–13). Meanwhile, other studies pointed out that PT and LBW are not related to DDE (14). The relationship between PT, LBW, and DDE remains inconclusive.

The World Health Organization (WHO) defines PT as living babies born less than 37 weeks of gestational age (15). Cortines et al. (16) found that 46.3% of PT has DDE in the primary dentition, which is 4.8 times higher than that of normal-born infants, and enamel hypoplasia (EHP) is the most common type, suggesting that the higher incidence rate of DDE in the primary dentition is severely related to PT. In 2012, a case–control study involving 80 children in Brazil showed that compared with normal-born infants, PT has a higher incidence rate of DDE, suggesting that PT is an important factor, which causes enamel opacity and EHP (17). Pinho et al. pointed out that the incidence rate of DDE is 15.3% in normal birth and 16.2% in PT, but there is no statistically significant difference in the incidence rate of DDE (12).

LBW is defined as a newborn less than 2,500 g at birth. Very low birth weight (VLBW) is defined as a newborn less than 1,500 g at birth (18). Previous studies reported that birth conditions are the common factor affecting DDE (10). Some researchers found that DDE is more common among LBW than that among normal birth weight (NBW) (19). On the contrary, Ruschel et al. (14) believed that the incidence rate of DDE is 11.3% in NBW and 12.5% in LBW, and the difference is not statistically significant.

Above all, the relationship between PT, LBW, and DDE remains controversial. This meta-analysis systematically analyzed the relationship between PT, LBW, and DDE in primary dentition, and provided a scientific and comprehensive basis for the prevention of DDE in primary dentition.

## Methods

### Focused question

This meta-analysis was conducted according to the guidance of Preferred Reporting Items for Systematic Reviews and Meta-Analyses (PRISMA) (Appendix 1) (20). Registered with PROSPERO, the registration number was CRD42021262761. Whether PT and LBW are more susceptible to DDE in the primary dentition than normal-born infants was explored, following the Participants, Intervention, Control, Outcome, Study (PICOS) design principle.

P: Children aged 0–6 years.

I: PT, LBW (including VLBW) or PT and LBW.

C: Full-term delivery or NBW.

O: DDE in the primary dentition.

S: Case–control study, cross-sectional study, and cohort study.

### Search strategy

Seven recognized electronic databases, PubMed, Wiley, Cochrane Library, Science Direct, China National Knowledge Infrastructure (CNKI), Database for Chinese Technical Periodicals (VIP), and WanFang, were retrieved for relevant publications in English or Chinese from inception up to June 2022, supplemented by manual retrieval. The relevant references of all retrieved articles were included. The data retrieval was through the combination of means of computer retrieval and manual retrieval of cross-sectional study, cohort study, and case–control study on the correlation between PT, LBW, and DDE. The relevant references of all retrieved articles were included. Medical subheadings (MeSH) combined with free word were applied to search through a computer: (“low birth weight” [MeSH] OR “very low birth weight” [MeSH]) AND (“preterm” [MeSH] OR “premature” [MeSH] OR “prematurity” [MeSH]) AND [“enamel development defect” OR “enamel hypoplasia” OR “Enamel opacity” (MeSH)] AND (“primary dentition” [MeSH] OR “deciduous dentition” [MeSH]).

### Inclusion criteria

Two reviewers independently identified and selected relevant studies by reading titles, abstracts, and full texts. The studies were selected based on the following inclusion criteria:

(1) Literature research types: Epidemiological research (case–control study, cohort study, cross-sectional study).
(2) Samples in the literature: Children less than or equal to 6 years old.
(3) Exposure factors in the literature: PT and LBW.
(4) Outcome index: DDE in the primary dentition in the literature and the modified DDE index published by FDI for the diagnostic criteria (1).
(5) Effective quantity: Odds ratio (OR) of DDE and 95% confidence interval (95% CI). All data which could be converted to OR were also included.

### Exclusion criteria

To reduce the selective bias, the study that met one of the following situations was excluded:

(1) Failure to check the credibility and the consistency.
(2) Not in English or Chinese.
(3) Repeatedly published literature.
(4) OR and 95% CI could not be extracted or transformed.

### Data extraction

Two reviewers (SX and LJ) selected the studies and extracted the data independently according to the inclusion and exclusion criteria. Disagreements were resolved through consensus or by seeking help from an arbitrator (HS). OR and 95% CI of DDE in PT, LBW, and VLBW compared with normal-born infants were calculated or extracted. The following information was extracted from each study: first author, year of publication, study method, number of patients, and age range of patients.

### Quality evaluation

In this study, the JBI scale was used to evaluate the quality of cross-sectional and cohort studies, and a score of 70% of full marks indicated a low risk of bias. The Newcastle–Ottawa Scale (NOS) was used to evaluate the quality of case–control studies, and 0–3, 4–6, and 7–9 points indicated low, moderate, and high quality, respectively.

### Statistical analysis

The meta-analysis was conducted by using the software STATA version 12.0 (STATA Corporation, College Station, TX, United States). The OR was used as the common measure of associated across studies. Heterogeneity across studies was assessed using the Cochrane Q Statistic (significance level at *P *< 0.10) and the *I*^2^ statistic (21). Heterogeneity was considered statistically insignificant if *P *> 0.10 and *I*^2^ ≤ 50%, and then the Mantel–Haenszel fixed-effect model (FEM) was used for calculating pooled OR among studies. Otherwise, the DerSimonian and Laird random-effect model (REM) was used for combining the results (22). Sensitivity analysis refers to the comparison between the combined effect after removing any one document and the result without removing it. The same overall result indicated that the results of this meta-analysis were stable and reliable. Publication bias was assessed by Begg's test for quantitative analysis with a *P *> 0.05 indicating statistical significance (23).

## Results

### Literature search and study characteristics

**Figure 1:**
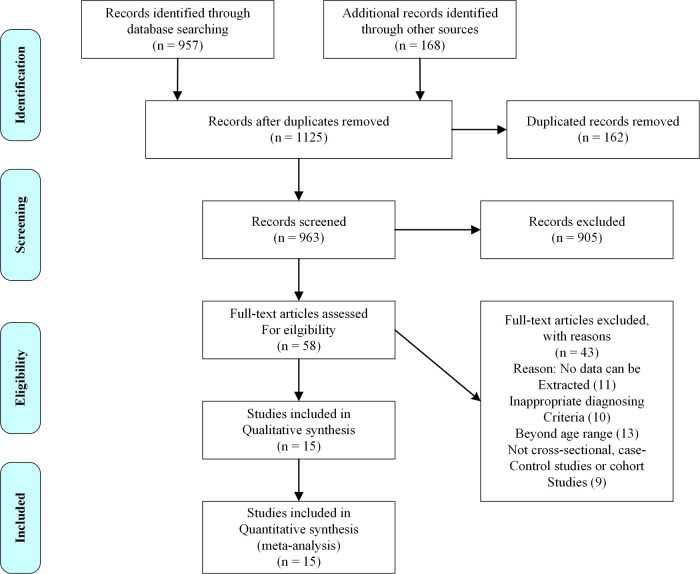
Flow chart of searching.

The flow diagram of the study selection process is shown in Figure 1. A total of 1,125 articles were identified according to the search strategy, of which 162 were excluded because of duplication. By screening the titles and abstracts, 905 articles were further excluded because they were review reports, which did not study the relationship between PT, LBW, and DDE. After screening the full text of the remaining articles, 43 articles were excluded because no efficient data could be extracted. At last, 15 articles containing 6,066 individuals were enrolled (3, 5, 14, 19, 24–34). Eight out of the 15 articles studied the relationship between PT and DDE. Seven articles studied the relationship between LBW and DDE, of which three studied the relationship between VLBW and DDE. The main characteristics of the selected studies for analysis are summarized in Table 1. Among the 15 articles, six studies were case–control studies, six studies were cross-sectional studies, and three studies were cohort studies. In addition, seven studies were listed according to the classification of DDE. Thirteen studies were of high quality and two studies were of moderate quality.

**Table 1:** The characteristics of the included studies.

Study ID	Age range	*n*	Case group (DDE/Health)			Control group (DDE/Health)			Study method	Subgroup of DDE	Quality score
			Preterm group	LBW group	VLBW group	Preterm group	LBW group	VLBW group			
Nelson, 2013	8 months	368			77/72			33/116	Cohort study	H:65 O:12	16
Nelson, 2013	18–20 months	378			98/86			89/95	Cohort study	H:60 O:38	16
Massoni, 2009	2–3 years	102		40/20			18/39		Cohort study		17
Takaoka, 2011	NA	91		39/6			20/26		Cohort study		16
Masumo, 2013	6–36 months	1221		18/32			76/154		Cross-section	H:11 O:7	17
Ruschel 2017	2–5 years	827		42/28			395/325		Cross-section	H:14 O:39	17
Wagner, 2017	3 years	377		3/13			17/344		Cross-section		18
Gabriela, 2017	2–3 years	467	12/60	6/38		49/327			Cross-section		17
Patricia, 2012	3–5 years	381	11/22	12/25		97/241	198/240		Cross-section		15
Masumo, 2014	6–36 months	816		10/76			96/934		Cross-section		17
Merglova, 2020	1 year	190		46/86	19/63		4/54	4/54	Case–control	H:36 O:14	7
Schüler, 2018	3–4 years	128	42/32			11/53			Case–control		5
Gravina, 2013	30–40 months	192	54/42	3/35	49/91	35/61	2/12	2/12	Case–control	H:36 O:18	7
Patricia, 2013	3–5 years	202	9/11	9/14		58/93	82/91		Case–control		7
Peres, 2015	3–5 years	204	21/10	8/5		74/99	87/104		Case–control		8
Franco, 2007	18–34 months	122	35/26			15/46			Case–control	H:48 O:32	5

DDE, developmental defects of enamel; LBW, low birth weight; VLBW, very low birth weight.

### Meta-analysis results

**Figure 2:**
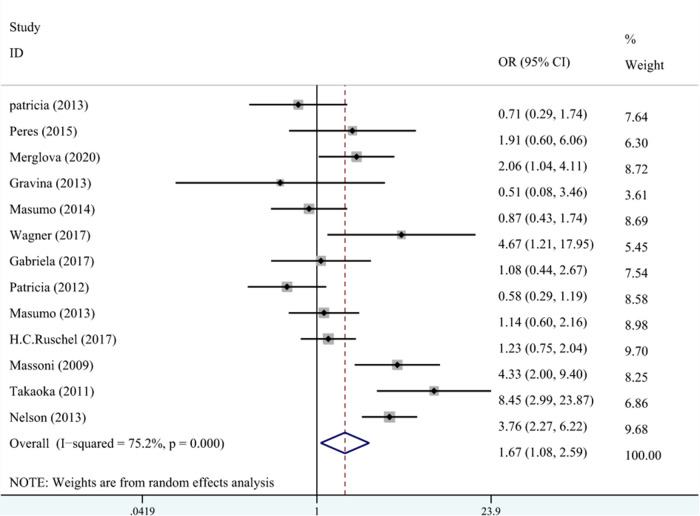
The forest plot shows the relationship between PT and DDE in primary teeth (REM). PT, preterm; DDE, developmental defects of enamel.

A total of eight studies on the relationship between PT and DDE are shown in Figure 2. The meta-analysis was conducted through the REM based on the result of heterogeneity (*I*^2 ^= 53.3%, *P*_heterogeneity_^ ^ = 0.036). The meta-analysis results of the included studies showed that PT was a risk factor for DDE compared with full-term infants (OR^ ^= 2.33, 95% CI: 1.55–3.51).

**Figure 3:**
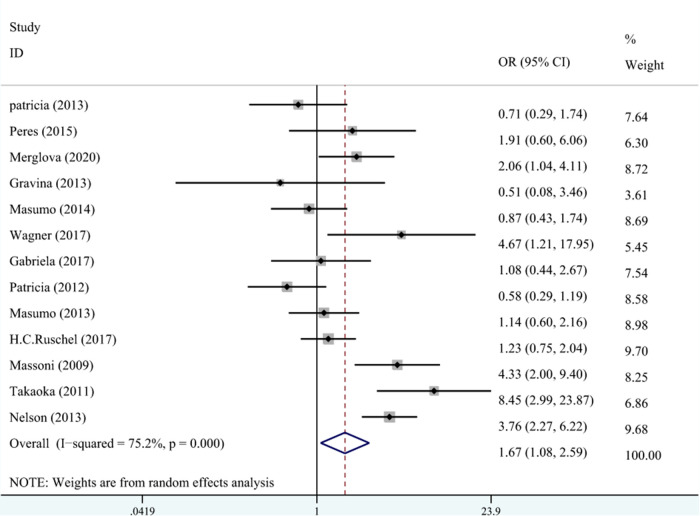
The forest plot shows the relationship between LBW and DDE (REM). LBW, low birth weight; DDE, developmental defects of enamel.

A total of 10 studies on the relationship between LBW and DDE are shown in Figure 3. The meta-analysis was conducted through the REM based on the result of heterogeneity (*I*^2 ^= 75.2%, *P*_heterogeneity_* *< 0.001). The results suggested that LBW was associated with DDE compared with NBW (OR^ ^= 1.67, 95% CI: 1.08–2.59).

**Figure 4:**
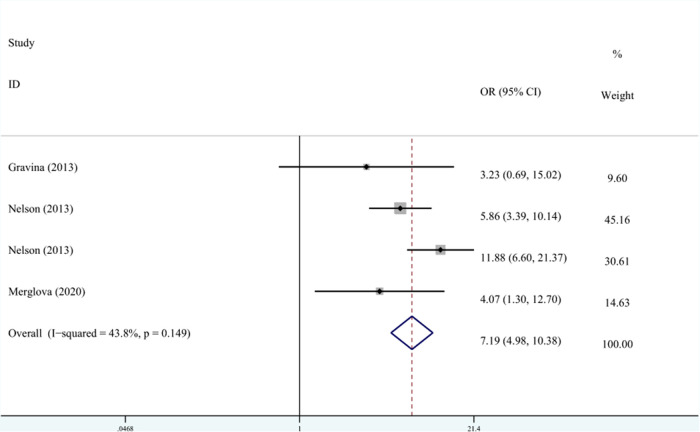
The forest plot shows the relationship between VLBW and DDE (FEM). VLBW, very low birth weight; DDE, developmental defects of enamel.

A total of three studies on the relationship between VLBW and DDE are shown in Figure 4. The meta-analysis was conducted through the FEM based on the result of heterogeneity (*I*^2 ^= 43.8%, *P*_heterogeneity_ = 0.149). The results suggested that VLBW was associated with DDE compared with NBW (OR^ ^= 7.19, 95% CI: 4.98–10.38).

### Sensitivity analysis

Sensitivity analysis was conducted by omitting one study at each time and recalculating the pooled results. Following the sensitivity analysis, the results of this study were stable and reliable. The results of this study were similar to the main results.

### Publication bias

There was no publication bias in the enrolled studies (PT and DDE: *P* = 0.75 > 0.05; LBW and DDE: *P* = 0.47 > 0.05; VLBW and DDE: *P* = 0.06 > 0.05).

### Subgroup analysis according to the classification of DDE in the study

(1) Relationship between PT and EHP

**Figure 5:**
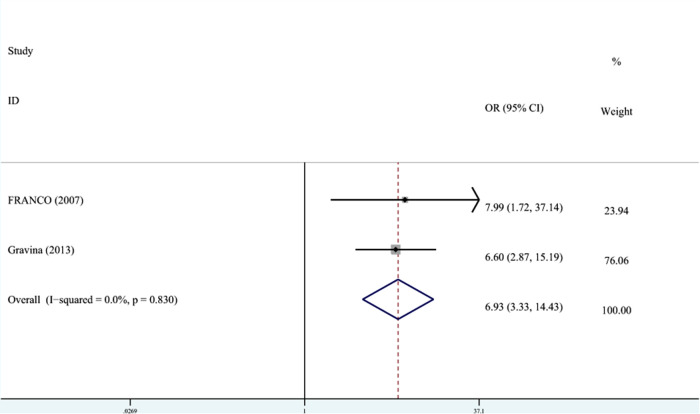
The forest plot shows the relationship between PT and EHP (FEM). PT, preterm; EHP, enamel hypoplasia.

DDE was divided into EHP and enamel opacity. Two studies recorded the relationship between PT and the classification of DDE. The meta-analysis was conducted through FEM (*I*^2 ^= 0%, *P*_heterogeneity_ = 0.83, OR^ ^= 6.89, 95% CI: 3.33–14.34). As shown in Figure 5, the OR combined with 95% CI horizontal line was on the right side of the dotted line, indicating that PT was associated with EHP.

**Figure 6:**
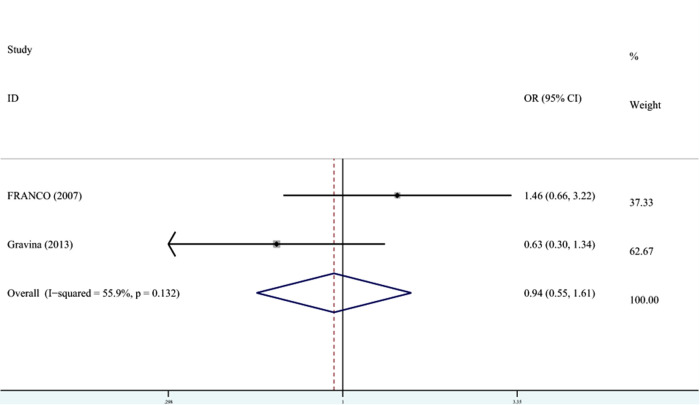
The forest plot shows the relationship between PT and enamel opacity (FEM). PT, preterm.

In terms of the relationship between PT and enamel opacity, the meta-analysis was conducted through REM (*I*^2 ^= 55.9%, *P*_heterogeneity_ = 0.13, OR^ ^= 0.94, 95% CI: 0.55–1.61). As shown in Figure 6, the OR combined 95% CI crossed the dotted line, indicating that PT was not associated with enamel opacity.

(2) Relationship between LBW and EHP

**Figure 7:**
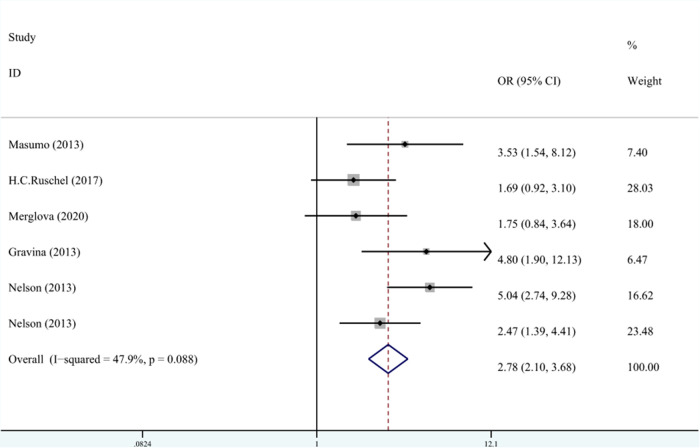
The forest plot shows the relationship between LBW and EHP (FEM). LBW, low birth weight; EHP, enamel hypoplasia.

Five studies recorded the relationship between LBW and the classification of DDE. The meta-analysis was conducted through FEM (*I*^2 ^= 47.9%, *P*_heterogeneity_ = 0.09, OR^ ^= 2.78, 95% CI: 2.10–3.68). As shown in Figure 7, LBW was associated with EHP.

**Figure 8:**
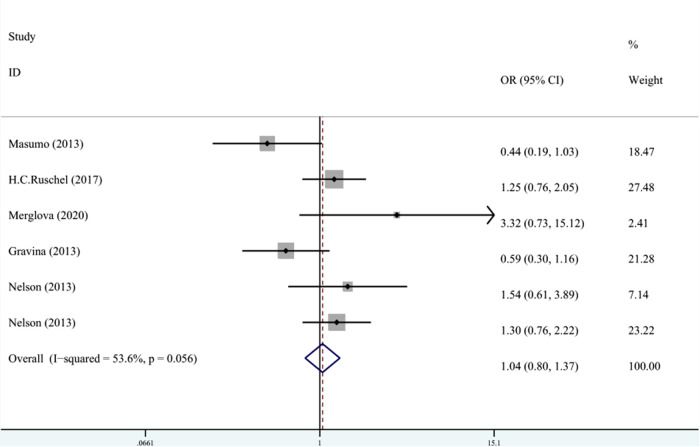
The forest plot shows the relationship between LBW and enamel opacity (REM). LBW, low birth weight.

In terms of the relationship between LBW and enamel opacity, the meta-analysis was conducted through REM (*I*^2 ^= 53.6%, *P*_heterogeneity_ = 0.06, OR^ ^= 1.03, 95% CI: 0.66–1.61). As shown in Figure 8, LBW was not associated with enamel opacity.

## Discussion

In this meta-analysis, a total of 15 articles about the relationship between PT, LBW, and DDE published before June 2022 were retrieved, involving 6,066 children aged 0–6 years old. Among the 15 studies, there were 6 cross-sectional studies, 6 case–control studies, and 3 cohort studies. The results showed that compared with full-term and NBW infants, PT and LBW were associated with a higher risk of DDE, and the incidence rate of EHP was higher both in PT and LBW. In previous studies, Jacobsen PE concluded a meta-analysis of 23 original studies on the relationship between PT and DDE from January 1966 to February 2013 (35). The results showed that the risk of DDE in PT is about 2.3 times that of full-term infants and that there is a strong correlation between PT and the risk of EHP in primary dentition. However, although 23 articles were included in this paper, a meta-analysis on high-quality studies and uniform diagnostic criteria was needed to increase credibility due to the lack of distinction between primary and permanent teeth, the huge difference in diagnostic criteria, the lack of research on the relationship between LBW and DDE, and the lack of research on the classification of DDE. This meta-analysis overcame the differences in diagnostic criteria in previous studies, focused on DDE in the primary dentition, and explored the relationship between PT, LBW, and DDE in the primary dentition. At the same time, the subgroup analysis was performed according to different types of studies and the classification of DDE to ensure the accuracy of this meta-analysis. Up to now, this was the most comprehensive systematic paper, which explored the association between PT, LBW, and DDE in the primary dentition.

DDE index introduced by the Federation Dentaire Internationale (FDI) divided DDE into demarcated opacity, diffused opacity, and EHP, which was used to quantify and grade DDE since 1982 (36). In 1992, FDI revised the DDE index into modified DDE (FDI 1992), which classified DDE into enamel opacity and EHP. Enamel opacity is characterized by the absence of defects on the enamel surface, but the presence of diffuse or well-defined areas of varying enamel translucency, which is classified as diffuse or localized opacity (37). Enamel opacity occurs during the calcification and maturation stages of enamel development, which is usually characterized by changes in translucency or enamel opacity, such as white, cream, yellow, or brown changes (38). Demarcated or diffused opacity is defined as enamel opacity (1). EHP involves the reduction of enamel thickness, which is a quantitative defect (38). EHP occurs during the enamel matrix formation stage, resulting in enamel changes and calcification disturbances due to damage to ameloblasts (37).

This meta-analysis showed that the prevalence of DDE in primary dentition was significantly associated with the physical condition of prenatal mothers and postpartum infants (38). Enamel was mainly composed of inorganic substances containing calcium and phosphorus. The accumulation of fetal calcium and phosphorus was mainly concentrated after 27 weeks of pregnancy, PT would cause insufficient fetal calcium and phosphorus storage and cause DDE (39). In addition, pregnant women were undernourished or metabolically deranged, the fetus cannot obtain adequate nutrition from the placenta, especially the deciduous teeth begin to mineralize at the 14th week of gestation and complete mineralization 1 year after the child was born, malnutrition in pregnant women during this period can lead to DDE in primary teeth (40). Previous studies have shown that smoking during pregnancy can cause PT and DDE (39). At the same time, nicotine affects the function of ameloblasts caused to DDE (39). The study showed that gestational diabetes mellitus during pregnancy could also cause PT and DDE in the primary dentition because the physiological disturbance, which was caused by excess glucose could contribute to the dysfunction of ameloblasts (41). A great deal of evidence showed that PT infants with impaired immune systems would increase the risk of DDE. In addition, PT infants were prone to respiratory diseases, cardiovascular diseases and other serious diseases, which would affect the development and mineralization of enamel (42). We should pay close attention to the situation of PT infants after birth to reduce the risk of DDE. Thus, paying attention to the physical condition of premature babies and mothers, and carrying out targeted prevention can help avoid DDE (39, 43).

The results of this meta-analysis showed that LBW infants were susceptible to DDE, which suggested that various unfavorable factors for the growth and development of LBW infants may be closely related to the occurrence of DDE. Cruvinel et al. believed that LBW infants are prone to hypoxia after birth, which can lead to various diseases. Hypoxia makes ameloblasts more sensitive and even causes damage to ameloblasts to affect the formation and mineralization of enamel, thus leading to DDE (17). In the case of hypoxia, various drugs are often used for treatment, and tracheal intubation or laryngoscope intubation is often conducted to overcome breathing difficulties (38). The excessive pressure exerted by the laryngoscope and tracheal intubation on the alveolar ridge can bring a negative influence, so trauma will permanently affect enamel development during this development period, resulting in DDE (44). Previous studies found the same result and pointed out that local trauma caused by left-sided tracheal intubation results in EHP mainly on the left side of the maxilla (12, 45). To sum up, the use of a laryngoscope and tracheal intubation in the treatment of hypoxia may be one of the causes of DDE.

The reasons for the heterogeneity in this meta-analysis included the selection bias of included samples and different clinical examination methods. There was a case–control study containing 128 children aged 3–4 years in this meta-analysis, of which the participants must get the consent of their mothers with a high level of education (24). In this study, infants were randomly selected from the expected date of confinement and invited to a dental clinic visit, which might contribute to heterogeneity. Additionally, the methods used to clinically detect DDE might also increase heterogeneity. There were three different examination methods in the literature: inspection, inspection + probing, and inspection + probing after cleaning. Visual examination alone was not as accurate as exploratory diagnosis of DDE, and examination of untreated tooth surfaces was not so accurate as visual examination alone after cleaning. Thus, inspection + probing could lead to an inaccurate result. In this meta-analysis, two studies were examined only by inspection, while the remaining 13 studies were examined by inspection + probing. Three studies did not treat the tooth surface before the examination, and the tooth surface was wiped using dry cotton balls in the remaining 10 studies. This study contained three research methods, i.e., six cross-sectional studies, three cohort studies, and six case–control studies. The heterogeneity was high in the relationship between PT, LBW, and DDE of all included studies. According to subgroup analysis, heterogeneity was low in each group, indicating that different research methods may be the main reason for the heterogeneity in the relationship between PT, LBW, and DDE.

There were several potential limitations of this study that deserved further consideration. (1) It was found that VLBW was highly correlated with DDE; unfortunately, there were only three related studies that met the inclusion criteria, which suggested that we should pay attention to the relationship between VLBW and DDE in the future. (2) In the included studies, the inspectors were all trained, but there were differences in the examination methods (inspection, inspection + probing, and inspection + probing after cleaning), which could influence the result. Therefore, studies with a high-consistency examination method are needed in the future. (3) In some of the included studies, some data were recorded and provided by hospital professionals with high accuracy, but in some studies, the data were provided by parents and guardians through memory, but there were deviations in the memory of different people, which could create information bias and affect accuracy. (4) All of the 15 studies in this meta-analysis were only in English and Chinese, and some studies in other languages that met the inclusion criteria might be lost, which would increase the limitations of this meta-analysis.

## Conclusion

This meta-analysis demonstrated that both PT and LBW especially VLBW increase the risk of DDE. In addition, both PT and LBW are associated with EHP, but their relationship with enamel opacity has not been verified.

## Data Availability

The original contributions presented in the study are included in the article/Supplementary Material, further inquiries can be directed to the corresponding author.

## References

[1] Report of an FDI Working Group. A review of the developmental defects of enamel index (DDE Index). Commission on oral health, research & epidemiology. Int Dent J. (1992) 42:411–26. 1286924

[2] Seow WK. Developmental defects of enamel and dentine: challenges for basic science research and clinical management. Aust Dent J. (2014) 59:143–54. 10.1111/adj.12104. 24164394

[3] Masumo R Bårdsen A Astrøm AN. Developmental defects of enamel in primary teeth and association with early life course events: a study of 6–36 month old children in Manyara, Tanzania. BMC Oral Health. (2013) 13:21. 10.1186/1472-6831-13-21. 23672512 PMC3671208

[4] Farsi N. Developmental enamel defects and their association with dental caries in preschoolers in Jeddah, Saudi Arabia. Oral Health Prev Dent. (2010) 8:85–92. 20480059

[5] Lunardelli SE Peres MA. Breast-feeding and other mother-child factors associated with developmental enamel defects in the primary teeth of Brazilian children. J Dent Child. (2006) 73:70–8. 16948367

[6] Folayan MO El Tantawi M Oginni AB Alade M Adeniyi A Finlayson TL. Malnutrition, enamel defects, and early childhood caries in preschool children in a sub-urban Nigeria population. PLoS One. (2020) 15(7):e0232998. 10.1371/journal.pone.0232998. 32609719 PMC7329100

[7] Liao Y Lin H. The prevalence and related factors of the devlomental defects of enamel in primary dentition. J Prevent Treat Stomatol Dis. (2002):188–9.

[8] Naidu RS Nunn JH. Prevalence of enamel developmental defects and relationship with early childhood caries in Trinidad. J Dent Child. (2016) 83(3):108–13. 28327259

[9] Salanitri S Seow WK. Developmental enamel defects in the primary dentition: aetiology and clinical management. Aust Dent J. (2013) 58:133–266. 10.1111/adj.12039. 23713631

[10] Hong J. Effect of preterm birth and low birth weight on children's oral development. World Latest Med Inf. (2016) 16:35–6.

[11] Seow WK. Enamel hypoplasia in the primary dentition: a review. ASDC J Dent Child. (1991) 58:441–52. 1783694

[12] Pinho JR Filho FL Thomaz EB Lamy ZC Libério SA Ferreira EB. Are low birth weight, intrauterine growth restriction, and preterm birth associated with enamel developmental defects? Pediatr Dent. (2012) 34:244–8. 22795159

[13] Aine L Backström MC Mäki R Kuusela AL Koivisto AM Ikonen RS Enamel defects in primary and permanent teeth of children born prematurely. J Oral Pathol Med. (2000) 29:403–9. 10.1034/j.1600-0714.2000.290806.x. 10972349

[14] Ruschel HC Vargas-Ferreira F Tovo MF Kramer PF Feldens CA. Developmental defects of enamel in primary teeth: highly prevalent, unevenly distributed in the oral cavity and not associated with birth weight. Eur Arch Paediatr Dent. (2019) 20:241–8. 10.1007/s40368-018-0402-4. 30888582

[15] World Health Organization. Preterm birth. (2022) Available online at: https: //www.who.int/news-room/fact-sheets/detail/preterm-birth (Accessed January 1, 2022).

[16] Cortines AAO Corrêa-Faria P Paulsson L Costa PS Costa LR. Developmental defects of enamel in the deciduous incisors of infants born preterm: prospective cohort. Oral Dis. (2019) 25:543–9. 10.1111/odi.13011 30537164

[17] Cruvinel VR Gravina DB Azevedo TD Rezende CS Bezerra AC Toledo OA. Prevalence of enamel defects and associated risk factors in both dentitions in preterm and full term born children. J Appl Oral Sci. (2012) 20:310–7. 10.1590/s1678-77572012000300003 22858696 PMC3881774

[18] Choi S Jung E Namgoong JM Jeong J Cha T Lee BS Extremely low birth weight infant surviving left congenital diaphragmatic hernia: a case report. Transl Pediatr. (2021) 10:3091–5. 10.21037/tp-21-355 34976775 PMC8649611

[19] Gravina DB Cruvinel VR Azevedo TD Toledo OA Bezerra AC. Enamel defects in the primary dentition of preterm and full-term children. J Clin Pediatr Dent. (2013) 37:391–5. 10.17796/jcpd.37.4.8q77717841781527 24046988

[20] Bernardo WM. PRISMA Statement and PROSPERO. Int Braz J Urol. (2017) 43:383–4. 10.1590/S1677-5538.IBJU.2017.03.02 28520335 PMC5462126

[21] Tiruneh C Gebremeskel T Necho M Teshome Y Teshome D Belete A. Birth prevalence of omphalocele and gastroschisis in sub-Saharan Africa: a systematic review and meta-analysis. SAGE Open Med. (2022) 10:20503121221125536. 10.1177/20503121221125536 36161211 PMC9500260

[22] DerSimonian R Laird N. Meta-analysis in clinical trials revisited. Contemp Clin Trials. (2015) 45:139–45. 10.1016/j.cct.2015.09.002 26343745 PMC4639420

[23] Xu Z Liu H Li S Han Z Chen J Liu X Palliative radiotherapy combined with stent insertion to relieve dysphagia in advanced esophageal carcinoma patients: a systematic review and meta-analysis. Front Oncol. (2022) 12:986828. 10.3389/fonc.2022.986828 36172146 PMC9511165

[24] Schüler IM Haberstroh S Dawczynski K Lehmann T Heinrich-Weltzien R. Dental caries and developmental defects of enamel in the primary dentition of preterm infants: case-control observational study. Caries Res. (2018) 52:22–31. 10.1159/000480124 29224001

[25] Merglova V Dort J. Developmental enamel defects of primary incisors in preterm infants with very low and extremely low birthweight. A case- control study. Eur J Paediatr Dent. (2020) 21:318–22. 10.23804/ejpd.2020.21.04.11 33337909

[26] Corrêa-Faria P Martins-Júnior PA Vieira-Andrade RG Marques LS Ramos-Jorge ML. Perinatal factors associated with developmental defects of enamel in primary teeth: a case-control study. Braz Oral Res. (2013) 27:363–8. 10.1590/s1806-83242013005000017 23689469

[27] Franco KM Line SR de Moura-Ribeiro MV. Prenatal and neonatal variables associated with enamel hypoplasia in deciduous teeth in low birth weight preterm infants. J Appl Oral Sci. (2007) 15:518–23. 10.1590/s1678-77572007000600012 19089191 PMC4327502

[28] Wagner Y. Developmental defects of enamel in primary teeth—findings of a regional German birth cohort study. BMC Oral Health. (2016) 17:10. 10.1186/s12903-016-0235-7 27430531 PMC4948106

[29] Pinto GDS Costa FDS Machado TV Hartwig A Pinheiro RT Goettems ML Early-life events and developmental defects of enamel in the primary dentition. Community Dent Oral Epidemiol. (2018) 46:511–7. 10.1111/cdoe.12408 30080266

[30] Masumo R Birungi N Bårdsen A Fadnes LT Astrøm AN. Impact of low birthweight on early childhood caries in 6–36 months old infants in Uganda: a cross-sectional study. Acta Odontol Scand. (2014) 72:312–20. 10.3109/00016357.2014.880189 24460034

[31] Nelson S Albert JM Geng C Curtan S Curtan S Miadich S Increased enamel hypoplasia and very low birthweight infants. J Dent Res. (2013) 92:788–94. 10.1177/0022034513497751 23857641 PMC3744269

[32] Takaoka LA Goulart AL Kopelman BI Weiler RM. Enamel defects in the complete primary dentition of children born at term and preterm. Pediatr Dent. (2011) 33:171–6. 21703068

[33] Popescu M Ionescu M Scrieciu M Popescu SM Mercuţ R Amărăscu MO Etiology study of acquired developmental defects of enamel and their association with dental caries in children between 3 and 19 years old from Dolj county, Romania. Children. (2022) 9(9):1386. 10.3390/children9091386 36138695 PMC9497921

[34] Jacobsen PE Haubek D Henriksen TB Østergaard JR Poulsen S. Developmental enamel defects in children born preterm: a systematic review. Eur J Oral Sci. (2014) 122:7–14. 10.1111/eos.12094 24164573

[35] An epidemiological index of developmental defects of dental enamel (DDE Index). Commission on oral health, research and epidemiology. Int Dent J. (1982) 32:159–67. 6956548

[36] Suckling GW. Developmental defects of enamel—historical and present-day perspectives of their pathogenesis. Adv Dent Res. (1989) 3:87–94. 10.1177/08959374890030022901 2701161

[37] Alaluusua S. Aetiology of molar-incisor hypomineralization: a systematic review. Eur Arch Paediatr Dent. (2010) 10:53–8. 10.1007/BF03262713 20403298

[38] Velló MA Martínez-Costa C Catalá M Fons J Brines J Guijarro-Martínez R. Prenatal and neonatal risk factors for the development of enamel defects in low birth weight children. Oral Dis. (2010) 16:257–62. 10.1111/j.1601-0825.2009.01629.x 19849806

[39] Neto MBC Silva-Souza KPD Maranhão VF Botelho KVG Heimer MV Dos Santos-Junior VE. Enamel defects in deciduous dentition and their association with the occurrence of adverse effects from pregnancy to early childhood. Oral Health Prev Dent. (2020) 18:741–6. 10.3290/j.ohpd.a45077 32895657 PMC12323744

[40] Embleton ND Berrington JE Dorling J Ewer AK Juszczak E Kirby JA Mechanisms affecting the gut of preterm infants in enteral feeding trials. Front Nutr. (2017) 4:14. 10.3389/fnut.2017.00014 28534028 PMC5420562

[41] Marler J Howland R Kimmons LA Mohrien K Vandigo JE Jones GM. Safety of propofol when used for rapid sequence intubation in septic patients: a multicenter cohort study. Hosp Pharm. (2022) 57(2):287–93. 10.1177/00185787211029547 35601715 PMC9117767

[42] Embleton ND Berrington JE Dorling J Ewer AK Juszczak E Kirby JA Mechanisms affecting the gut of preterm infants in enteral feeding trials. Front Nutr. (2017) 4:14. 10.3389/fnut.2017.00014 28534028 PMC5420562

[43] Caufield PW Li Y Bromage TG. Hypoplasia-associated severe early childhood caries—a proposed definition. J Dent Res. (2012) 91:544–50. 10.1177/0022034512444929. 22529242 PMC3348067

[44] Merheb R Arumugam C Lee W Neonatal Serum phosphorus levels and enamel defects in very low birth weight infants. JPEN J Parenter Enteral Nutr. (2016) 40:835–41. 10.1177/0148607115573999 25733338

[45] Alaluusua S. Aetiology of molar-incisor hypomineralisation: a systematic review. Eur Arch Paediatr Dent. (2010) 11:53–8. 10.1007/BF03262713 20403298

